# Supplementary material for: Comprehensive Annotation and Functional Exploration of MicroRNAs in Lettuce
Source: Front Plant Sci. 2021 Dec 24;12:781836. doi: 10.3389/fpls.2021.781836 (PMC8739914; doi:10.3389/fpls.2021.781836)
Supplement: Supplementary file 3 [file Data_Sheet_9.ZIP › GSM2230753.plot/Lsa-miRN1655_Lsat_1_v5_gn_9_121380.1_1141_TPlot.pdf]

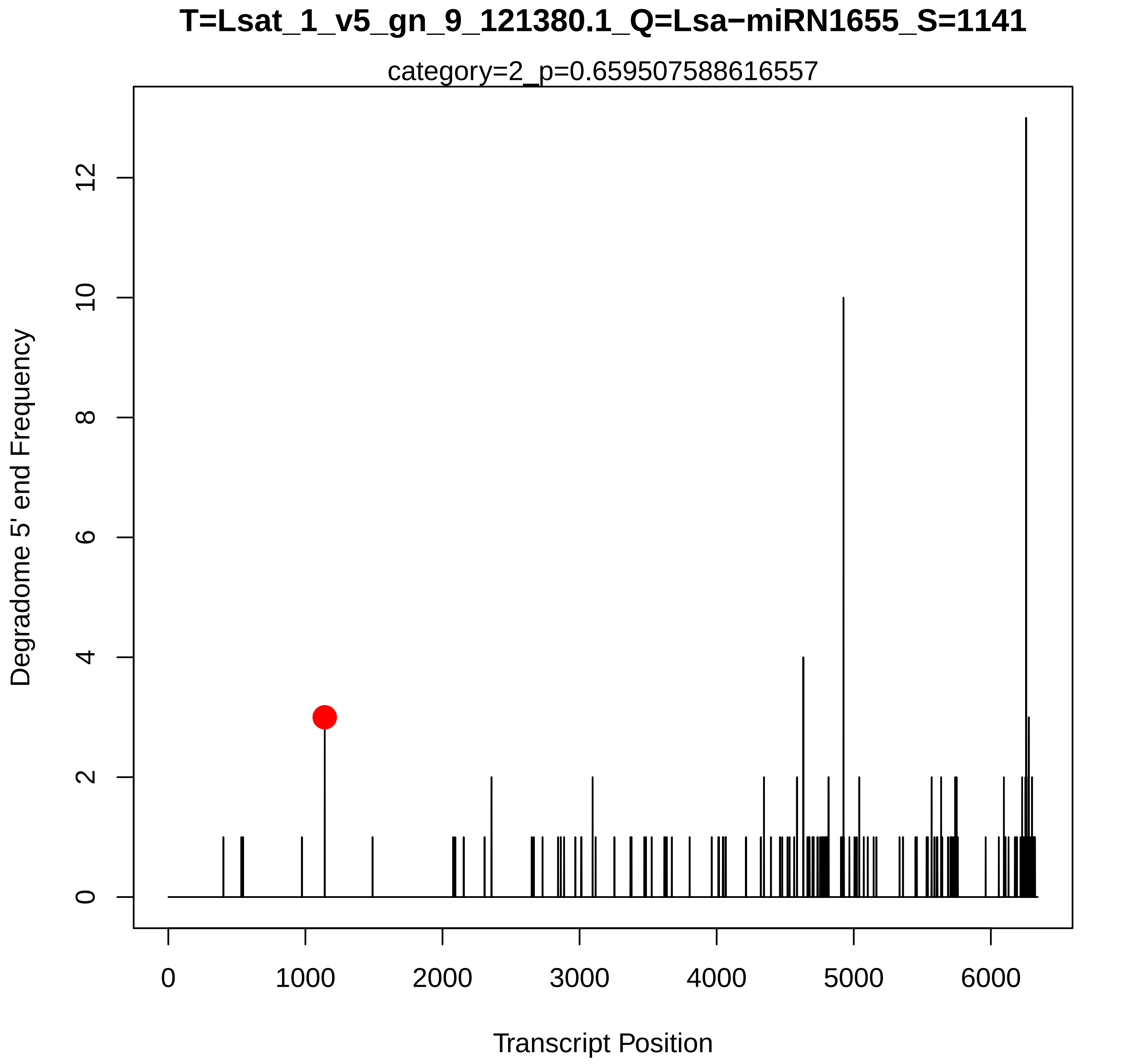

T=Lsat_1_v5_gn_9_121380.1_Q=Lsa−miRN1655_S=1141
category=2_p=0.659507588616557
Degradome 5' end Frequency
0
2
4
6
8
10
12
0
1000
2000
3000
4000
5000
6000
Transcript Position
